# Genesis and development processes of fractures in granite: Petrographic indicators of hydrothermal alteration

**DOI:** 10.1371/journal.pone.0251198

**Published:** 2021-05-04

**Authors:** Takashi Yuguchi, Yuya Izumino, Eiji Sasao

**Affiliations:** 1 Faculty of Science, Yamagata University, Yamagata, Japan; 2 Japan Atomic Energy Agency, Toki, Gifu, Japan; University of Science and Technology Beijing, CHINA

## Abstract

Biotites occur with varying degrees of alteration within a granite. This study analyzes the relationships among alteration indicators, areal microvoid fractions in chloritized biotite, and macroscopic fracture frequencies in the Toki granite, central Japan, to establish the genesis and development processes of fractures in granite. Appropriate characterizations for the frequency distribution of macroscopic fractures in granite can assist in understanding potential hydrogeological applications, which contributes to safety evaluations for geological disposal and storage. Borehole 06MI03, drilled to a depth of 191 m, was used to obtain samples for the analysis. In total, 24 samples that depicted variations in the macroscopic fracture frequency were selected. Petrographic alteration indicators using biotite chloritization as innovative methods are proposed to evaluate the extent of hydrothermal alteration and fracture frequency within granites. The alteration indicators are defined as the ratio between the alteration product area and the original mineral area. Furthermore, the volume of microscopic fractures and micropores in the mineral was quantitatively characterized by the areal fraction of microvoids in minerals through image analysis. Samples with high macroscopic fracture frequencies correspond to a high number of areal microvoid fractions and large alteration indicators. Microvoids, which are the source of macroscopic fractures, occurred at temperatures between 350 and 780°C and can be evaluated by intrinsic factors, such as alteration indicators. Subsequent faulting and unloading (extrinsic factors) developed microvoids into macroscopic fractures. Intrinsic factors are used to evaluate the source of macroscopic fractures, and therefore contribute to the characterization of present and future distributions of macroscopic fracture frequencies.

## Introduction

**Fig 1:**
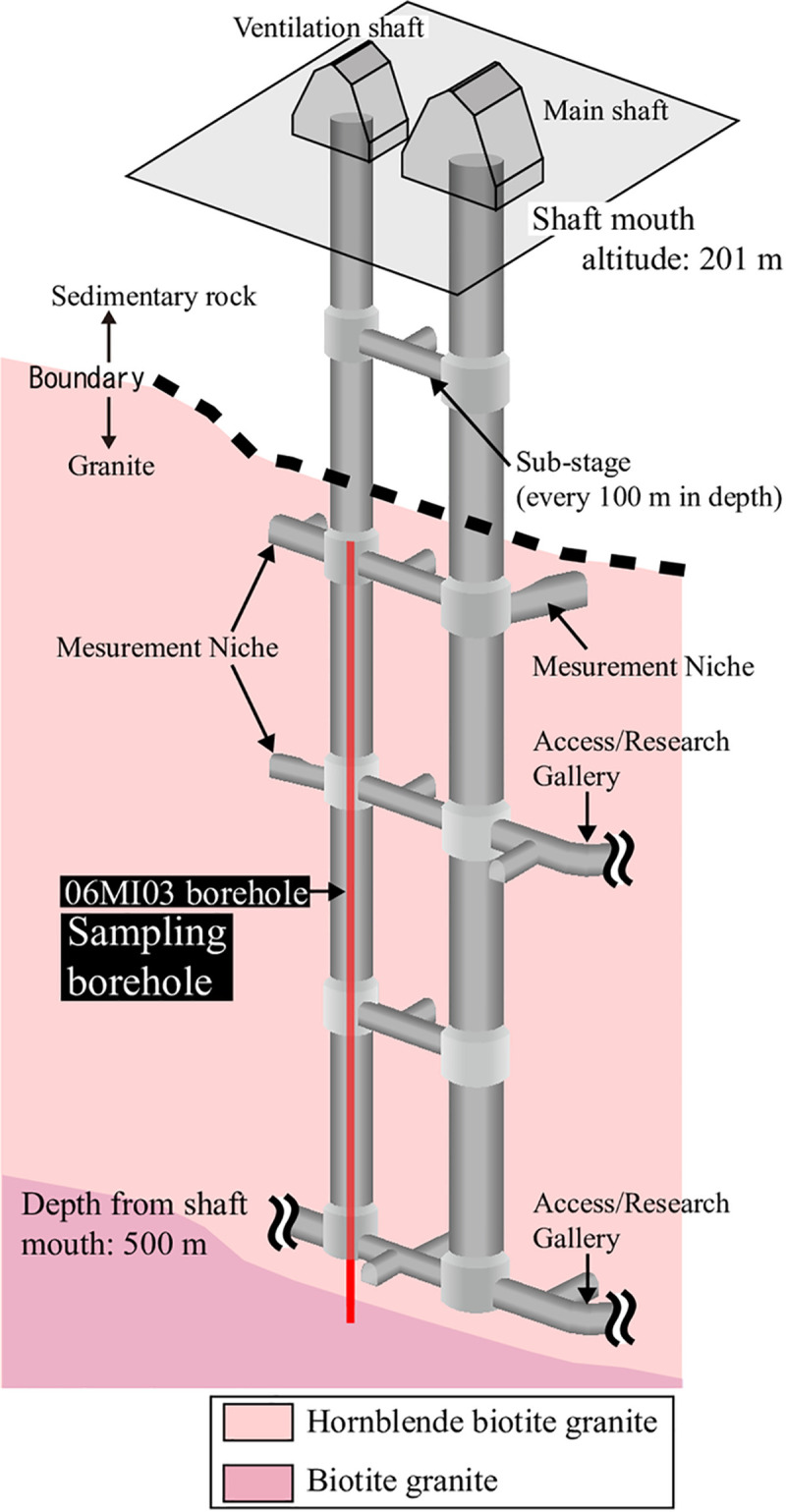
Mizunami Underground Research Laboratory (MURL). Schematic overview of the shafts in the MURL and borehole 06MI03, along with the distribution of hornblende-biotite granite and biotite granite in the lithofacies. Shafts at a depth of approximately 170 m intersect the unconformity between the Mizunami Group and the Toki granite.

Operability assessments of geological nuclear waste disposal and oil and natural gas storage in crystalline (granitic) rocks are currently conducted in several countries (e.g., Sweden, Finland, and Japan). In geological disposal, high-level nuclear waste must be fully segregated from aboveground human society during the period that it will be characterized by dangerous levels of radiation. In geological storage, oil and natural gas must be immobilized within the chamber in the granitic rock mass until their use. Mass transfer characterization within granite contributes to safety evaluations for geological disposal and storage. In an orogenic area such as the Japan Arc, fractures and small faults inevitably occur in granitic rocks and act as fluid flow and contaminant transport conduits [1–3]. Therefore, hydrogeological applications of granite require appropriate fracture distribution, frequency, and network characterization [3–7]. The petrographic study of a granitic rock mass is an appropriate subject to develop an understanding of fracture characteristics [e.g. 5, 8–15]. In a previous study on the analysis of fracture distribution, Takagi et al. [16] described the distribution of open microfractures in granitic minerals based on polarized light microscope and cathodoluminescence images. Mazurek [8] showed that the fracture frequency in granite changes depending on differences in the rock facies. Chigira [9] proposed that the spacing of micro-sheeting is correlated with the grain size of the constituent minerals. Although Mazurek [8] and Chigira [9] demonstrated the relation between fracture frequency and petrography in granite, these studies have not explained the fracture genesis and development process in granite. This study presents a new petrological method to evaluate the extent of hydrothermal alteration while revealing the fracture genesis and development using the Mizunami Underground Research Laboratory (MURL) in central Japan (Fig 1) as an example, thereby contributing to effective fracture frequency distribution characterization.

**Fig 2:**
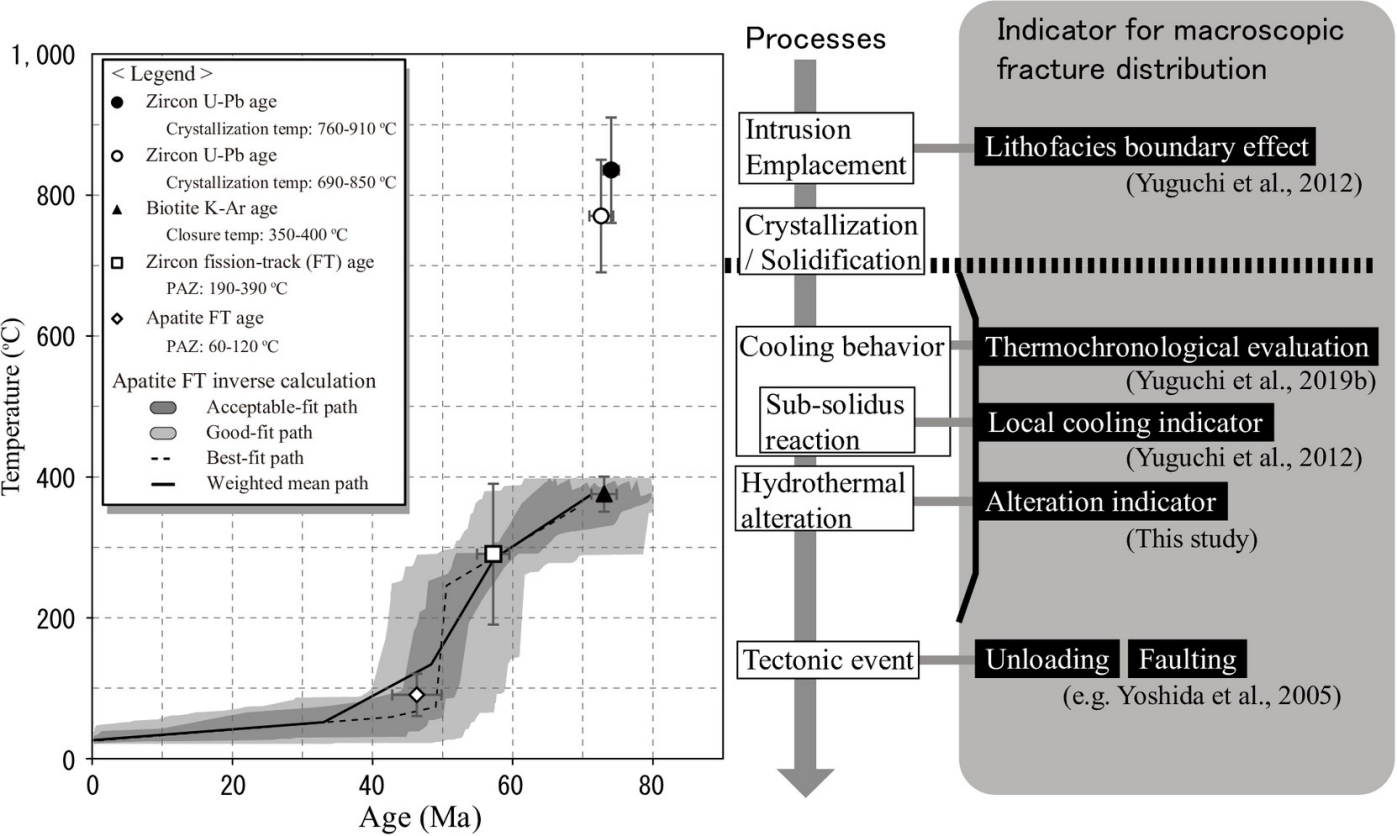
Time-temperature (*t-T*) paths of sample DH2 RA03 (–302.1 masl in borehole DH2) from the Toki granite [21], along with the intrinsic and extrinsic factors for an informative evaluation of the fracture population in a granite. PAZ indicates the partial annealing zone.

Large-scale fractures visible in borehole television (BTV) data are defined as macroscopic fractures in this study, which contribute to prompt mass transfer within the granite [6, 7, 17]. Macroscopic fractures in granites are caused by a combination of intrinsic factors associated with thermal stress and extrinsic factors associated with tectonic faulting and unloading [7, 18]. Petrographic characterizations described by Mazurek [8] and Chigira [9] correspond to intrinsic factors. Thermal stress is induced by differential contraction during the cooling of a pluton [7, 18–20]. Bergbauer and Martel [18] predicted thermal stress based on a thermo-mechanical stress analysis assuming two-dimensional conductive cooling for the Lake Edison Granodiorite, California. The modeled orientations of the most compressive thermal stress trajectories within the pluton were consistent with the strike of the macroscopic fractures, suggesting that thermal stress can contribute to the formation of fractures in a pluton. The evaluation of the fracture distribution is based on parameters that reflect the thermal stress within granite: the local cooling indicator (LCI) and the thermochronological evaluation [5, 21]. The LCI is deduced from the development of sub-solidus reaction textures [5], which occur at the exsolution (690–780°C) and deuteric (below 500°C) stages during cooling (Fig 2). The thermochronological evaluation corresponds to the thermal conditions from crystallization/solidification (approximately 780°C) to biotite K–Ar closure (350–400°C) [21].

This study focuses on hydrothermal alteration as the petrological phenomena at thermal conditions below the biotite K–Ar closure temperature, i.e., the thermal conditions that are not used to evaluate the fracture frequency distribution. Hydrothermal alteration of the Toki granite progressed through the following successive processes: 1) biotite chloritization, 2) plagioclase alteration, and 3) precipitation of carbonate minerals [22]. The alteration processes of biotite chloritization and plagioclase alteration occurred at temperatures of 180–350°C within the rock body [23]. To evaluate the fracture frequency distribution, this study presents a methodology using the alteration indicators of biotite chloritization to quantitatively characterize the extent of hydrothermal alteration in mineral and rock samples (see Materials and Methods). We refer to the microscopic fractures (microcracks) and micropores within minerals that are visible via polarization microscopy and electron scanning microscopy as microvoids. This study also focuses on the volume of microvoids in the chloritized biotite because the hydrothermal alteration of granitic rock is mainly constrained by dissolution–precipitation processes during the penetration of hydrothermal fluids through microscopic fractures and micropores within minerals [23–25]. The volume of microscopic fractures and micropores in chloritized biotite was quantitatively characterized based on the areal fraction of microvoids in minerals through image analysis (see Materials and Methods, Fig 4). Previous studies have not reported on the methodologies that characterize the 1) alteration extent and 2) areal fraction of microvoids; thus, the technical development of this methods will be innovative for a similar area characterized by hydrothermal alteration.

A comparison of the areal fractions of microvoids in minerals with the alteration indicators provides insight into the factors constraining the developmental extent of hydrothermal alteration. Furthermore, we deduce new knowledge on the genesis and development processes of fractures in granite from the relationships among the areal fractions of microvoids in minerals, alteration indicators, and macroscopic fracture frequencies. The Toki granite represents an ideal example for this study. Two 500 m-long vertical shafts penetrated the Toki granite to allow for the extraction of fresh samples of variably fractured granite. In particular, biotite chloritization occurred throughout the rock body; Yuguchi et al. [23] describes their formation mechanisms in detail.

## Materials and methods

### Toki granite and the Mizunami Underground Research Laboratory

The Toki granite in the Tono district, central Japan, is one of the Late Cretaceous plutonic bodies of the Sanyo Belt [26]. The Toki granite is a stock, approximately 14 × 12 km^2^ in areal extent [27], comprising a zoned pluton with three lithofacies grading from muscovite-biotite granite at the margin through hornblende-biotite granite to biotite granite in the interior [28]. The Tsukiyoshi Fault occurs within the Toki granite. This fault strikes E–W and dips 65–75° to the south [29]. The petrography and geochronology of the Toki granite are described in detail in Yuguchi et al. [5, 21, 30–32].

The MURL, which consists of two vertical shafts (main and ventilation shafts) (Fig 1), is located in the sedimentary Mizunami Group, which unconformably overlies the Toki granite (Fig 1; [33, 34]). The main and ventilation shafts are 500 m deep and range from an elevation of 201 masl (meters above sea level) (ground level) to an elevation of –299 masl (shaft bottom) (Fig 1). In the MURL, the fault along the main shaft (referred to as the main shaft fault) has a strike of 30° N to 40° W and a dip of 80° SW [35].

### Sample description and fractures

This study used borehole 06MI03 (vertical section is 336-m long and the diameter is 123 mm) (Fig 1) to obtain samples for analysis. Borehole 06MI03 was drilled to a depth of 191 m in the ventilation shaft before the shaft was excavated below 191 m (Fig 1). Two geostructural domains, i.e., an upper highly fractured domain (UHFD) and a lower sparsely fractured domain (LSFD), have been identified in the Toki granite based on macroscopic fracture frequency [36]. In borehole 06MI03, the boundary between the UHFD and LSFD is at a depth of approximately 265 m. BTV investigation provided images of 733 macroscopic fractures from borehole 06MI03 (S1 Table), with macroscopic fracture frequencies ranging from 0 to 48 fractures per 5-m interval [37, 38]. Twenty-four samples with variations in macroscopic fracture frequency were selected for this study (S1 Table). Samples No. A1–A12 were collected from intervals in the UHFD ranging from 3 fractures per 5 m (No. A12) to 48 fractures per 5 m (No. A1). Samples No. 1–12 were collected from intervals in the LSFD ranging from 0 fractures per 5 m (No. 1, 2, 7, and 12) to 16 fractures per 5 m (No. 9) (S2 Table).

**Fig 3:**
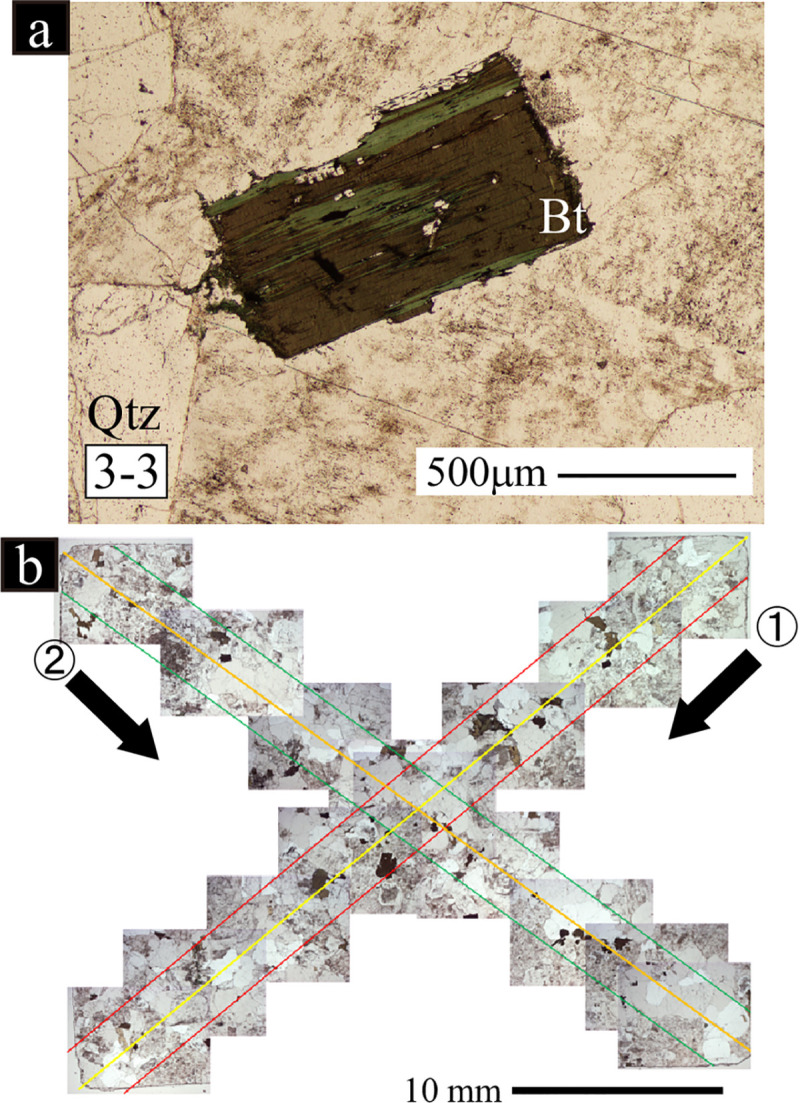
Polarizing microscope images of the biotite chloritization (a: sample No. 3–3) and an example of the thin section images used for the selection of target minerals (b). Two lines, the 1^st^ and 2^nd^ traverses, were established diagonally across a thin section. The traverses were 3.0 mm wide for biotite chloritization selections. The 1^st^ traverse for the biotite chloritization indicator selection is indicated by the area between the red lines and the 2^nd^ traverse is the area between the green lines. Bt: biotite; Qtz: quartz.

**Fig 4:**
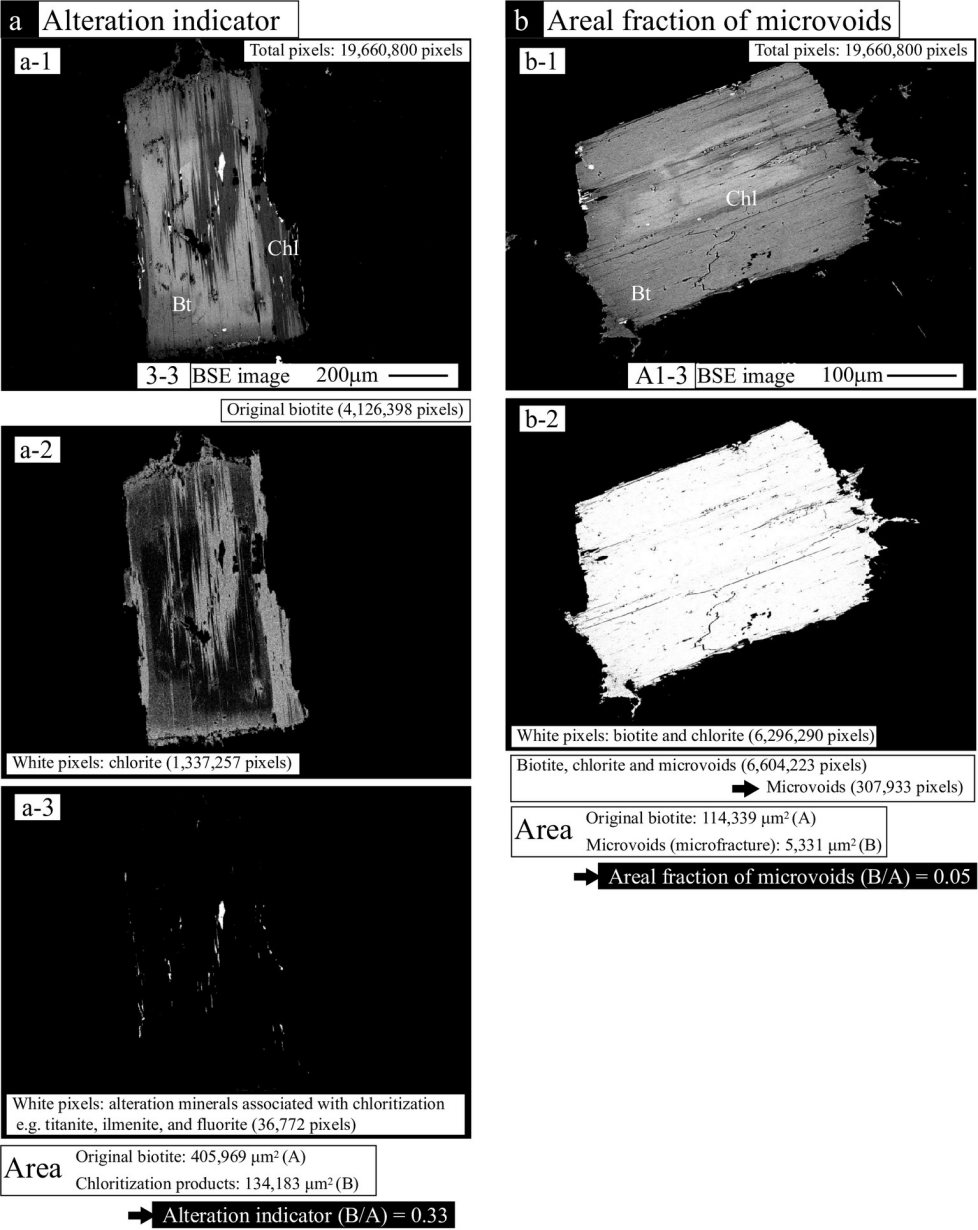
Example images showing the processing technique using Adobe Photoshop® to reveal the alteration indicator (a) and the areal fraction of microvoids (microscopic fractures and micropores) in the chloritized biotites (b).

Petrographic analysis demonstrates that biotites occur ubiquitously throughout the samples and are accompanied by chloritization (Fig 3A). The chloritization is also characterized by some associated minerals, such as titanite, ilmenite, K-feldspar, and fluorite [23]. Thus, samples with hydrothermal alteration from intervals with different macroscopic fracture frequencies are suitable for this study. As a control experiment, samples No. 1 and 7 were collected from the same depth range (307.7–312.7 m) to evaluate similarities or differences in the alteration indicators and areal microvoid fractions. The microvoids within the chloritized biotite consist of microscopic fractures and micropores. Linear microscopic fractures are less than 5 μm in width and are interconnected. Micropores have columnar and rounded shapes less than 7 μm in size along their principal axes. The chloritized biotite is typically accompanied by microscopic fractures, which predominately occur along cleavages (Fig 4A and 4B) rather than micropores.

### Alteration indicator

For individual minerals, the alteration indicator was obtained by the following work sequence: 1) selection of the target minerals in the rock sample, 2) acquisition of backscattered electron (BSE) images, and 3) image analysis. Petrographic data were obtained from the thin sections. The thin sections were prepared carefully to prevent mineral detachment. Twenty chloritized (unaltered) biotites were employed fundamentally as the target quantities in the thin sections. Selection of target minerals was conducted using polarizing microscopy. Two diagonal lines were established in the thin sections, i.e., the 1^st^ and 2^nd^ traverses (Fig 3B). The widths of the traverses were 3.0 mm. All biotites intersected by the 1^st^ traverse were targeted. If less than 20 biotites were identified in the 1^st^ traverse, additional minerals were obtained from the 2^nd^ traverse until the target quantity (N = 20) was achieved (Fig 3B).

The BSE images of the target minerals were collected using a JEOL IT100A scanning electron microscope at Yamagata University, operating at an accelerating voltage of 15 kV and a beam current of 1.5 nA. The areas (pixels) of the individual minerals and corresponding alteration areas were determined using the Adobe Photoshop® image processing software. Fig 4A shows the area of biotite chloritization (sample No. 3–3), including biotite as a reactant and chlorite, titanite, ilmenite, and fluorite as the alteration products (Fig 4A-1), which correspond to the area of the original (magmatic) biotite. The image was binarized with white pixels corresponding to the mineral (Fig 4A-2) and black pixels for the remainder of the sample. The areas of the associated alteration products were also counted by white pixels in a binarized image (Fig 4A-3). The pixels were converted into areas using the scale of the image. The alteration indicator is given by the total area of the alteration products divided by the area of the original biotite (Fig 4A). The alteration indicators of biotite chloritization in the range of 0–1 represent the extent of hydrothermal alteration, where relatively weak alteration is indicated by values closer to zero and relatively strong alteration by values close to 1. During the production of thin sections, the cutting surface of the mineral becomes random, such that it cannot be guaranteed to provide a cross-section that passes through the identical parts (e.g., core parts) of the mineral. This may yield variations in the alteration indicator in the minerals. Therefore, the alteration indicator for a rock sample is represented by the mean value and standard deviation of the indicators for the target minerals.

### Areal fraction of microvoids in minerals

For individual minerals, the areal fraction of microscopic fractures was obtained by BSE image analysis of the chloritized biotites accompanied by an alteration indicator. The areas (pixels) of the individual minerals and corresponding microvoid areas were determined using the Adobe Photoshop® image processing software. Fig 4B shows the area of biotite chloritization (sample No. A1-3: Fig 4B-1), which corresponds to the area of the original (magmatic) biotite. The image was binarized with white pixels corresponding to the mineral (biotite and alteration products, such as chlorite, titanite chlorite, and titanite in Fig 4B-2) and black pixels corresponding to microvoid areas within the mineral and the area surrounding the mineral. The pixels were converted into areas using the scale of the image, and the areal microvoid fractions were obtained by dividing the total area of the microvoids by the area of the original biotite (Fig 4B). The areal fraction of microscopic fractures and micropores in chloritized biotite is represented in the range of 0–1, where a relatively small area is indicated by values closer to zero and a relatively large area by values closer to 1. The areal fraction of the microvoids in a rock sample is represented by the mean value and standard deviation of the areal fractions for the target minerals.

## Results

### Alteration indicators for biotite chloritization

The mean values for the biotite chloritization indicator range from 0.13 to 0.74 (N = 24: S2 and S3 Tables). In the control experiment with samples No. 1 and 7, the biotite chloritization alteration indicator in sample No. 1 had a mean of 0.33 and a standard deviation of 0.12. Sample No. 7 had a mean of 0.22 and a standard deviation of 0.08 (S2 Table). The standard deviation range of sample No. 1 overlaps with that of sample No. 7. Thus, two different samples collected from the same depth range show similar alteration indicator values, implying the relevance of the indicators and methodology when evaluating the extent of hydrothermal alteration in a rock sample.

### Areal fractions of microvoids in the chloritized biotites

The mean values for the areal fractions of microvoids in the chloritized biotites range from 0.02 to 0.11 (N = 24: S2 and S4 Tables). In the control experiment with samples No. 1 and 7, the mean areal fractions of microvoids in sample No. 1 had a mean of 0.08 with a standard deviation of 0.03. Sample No. 7 had a mean of 0.06 with a standard deviation of 0.02 (S2 Table). The standard deviation range of sample No. 1 overlaps with that of sample No. 7, implying that they represent similar areal fractions of microvoids.

## Discussion

### Relationship between alteration indicators and macroscopic fractures

**Fig 5:**
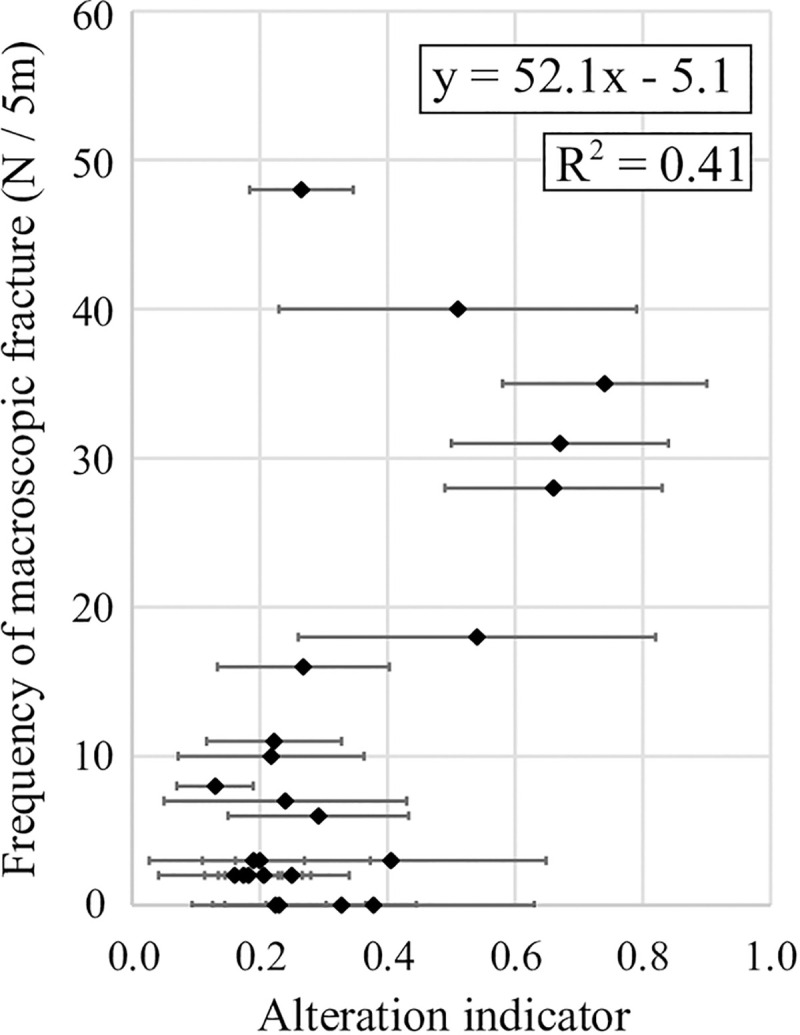
Relationship between the alteration indicators for rock samples and the macroscopic fracture frequency.

Fig 5 shows the relationship between the alteration indicators of biotite chloritization and the macroscopic fracture frequency at the same depth intervals. Small indicators correspond to low macroscopic fracture frequencies while large indicators correspond to high fracture frequencies and demonstrate positive correlations (*R*^2^ = 0.41). The regression lines are expressed as y = 52.1x – 5.1 (Fig 5), where y is the macroscopic fracture frequency and x is the alteration indicator. Such positive correlations indicate that the development of hydrothermal alteration appears to be related to the developmental factor of macroscopic fractures. However, sample No. A1, which has a macroscopic fracture frequency of 48 fractures per 5 m, has low alteration indicators; this is a notable exception to the positive correlations observed.

### Strikes and dips of macroscopic fractures

**Fig 6:**
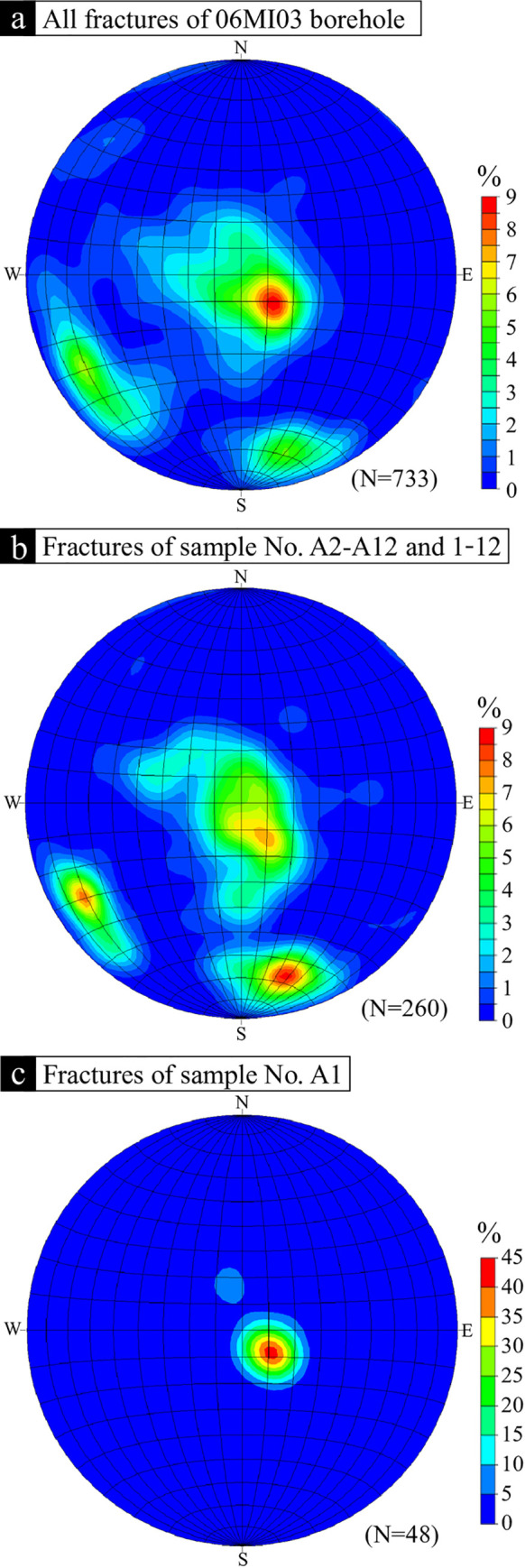
Schmidt stereonet diagrams showing the fracture orientations in borehole 06MI03 (a), in samples No. A2–A12 and 1–12 (b), and in sample No. A1 (c). The data are shown as concentrations of the poles to the fracture planes. The stereonets are lower-hemisphere projections.

Macroscopic fracture orientations were extracted from the BTV data [37, 38]. Observed fractures were categorized into three groups according to angle: low-angle (≦30° dip), intermediate-angle (30° to 60° dip), and high-angle (≧60° dip) fractures. The fracture orientations in borehole 06MI03 (Fig 6A), in samples No. A2–A12 and 1–12 (Fig 6B), and in sample No. A1 (Fig 6C) are shown as concentration distributions on Schmidt stereonet diagrams.

Borehole 06MI03 is characterized by low-angle and high-angle fractures with NW–SE and E–W strikes and southerly dips (N = 733: Fig 6A). The fractures of samples No. A2–A12 and 1–12 show similar orientations (N = 260: Fig 6B). In sample No. A1, however, low-angle fractures are predominant (N = 48: Fig 6C). Due to the vertical borehole trajectory, high-angle fractures will be under-sampled relative to low-angle fractures [39, 40]. Considering borehole bias, three main macroscopic fracture orientations are apparent: low-angle fractures, high-angle fractures with NW–SE strikes, and high-angle fractures with E–W strikes. The low-angle fractures observed in the sample developed from the response of the rock to unloading during granite exhumation [17, 41]. The high-angle fractures that strike E–W are parallel to the Tsukiyoshi Fault, and those that strike NW–SE are parallel to the main shaft fault (Fig 6C). Therefore, the macroscopic fracture orientations in the study area are controlled by unloading and faulting. Moreover, the frequency of macroscopic fracture can be accounted for by the alteration indicators.

The predominantly low-angle fractures (Fig 6C) in sample A1 indicate that this sample was individually and strongly influenced by unloading, which corresponds to the extrinsic factors evident. Sample No. A1 corresponds to a range from 208.67–213.67 m in depth from ground level (S1 Table) and is the shallowest (i.e., the highest elevation) of the rock samples. The unloading caused a development of low-angle fractures due to the removal of upper rock mass during granite exhumation [17, 41]. When the upper rock mass was removed, the deeper parts received a larger weight than the shallow parts. Thus, previous studies have reported on the dominant occurrence of low-angle fractures at shallower depths in borehole 06MI03 [37, 38]. These observations are consistent with the significant influence of unloading for sample A1 and indicate that the fracture frequency in the rock mass at shallow depths is due to unloading as the extrinsic factor and cannot be evaluated using alteration indicators.

### Relationship between microvoids and alteration indicators

**Fig 7:**
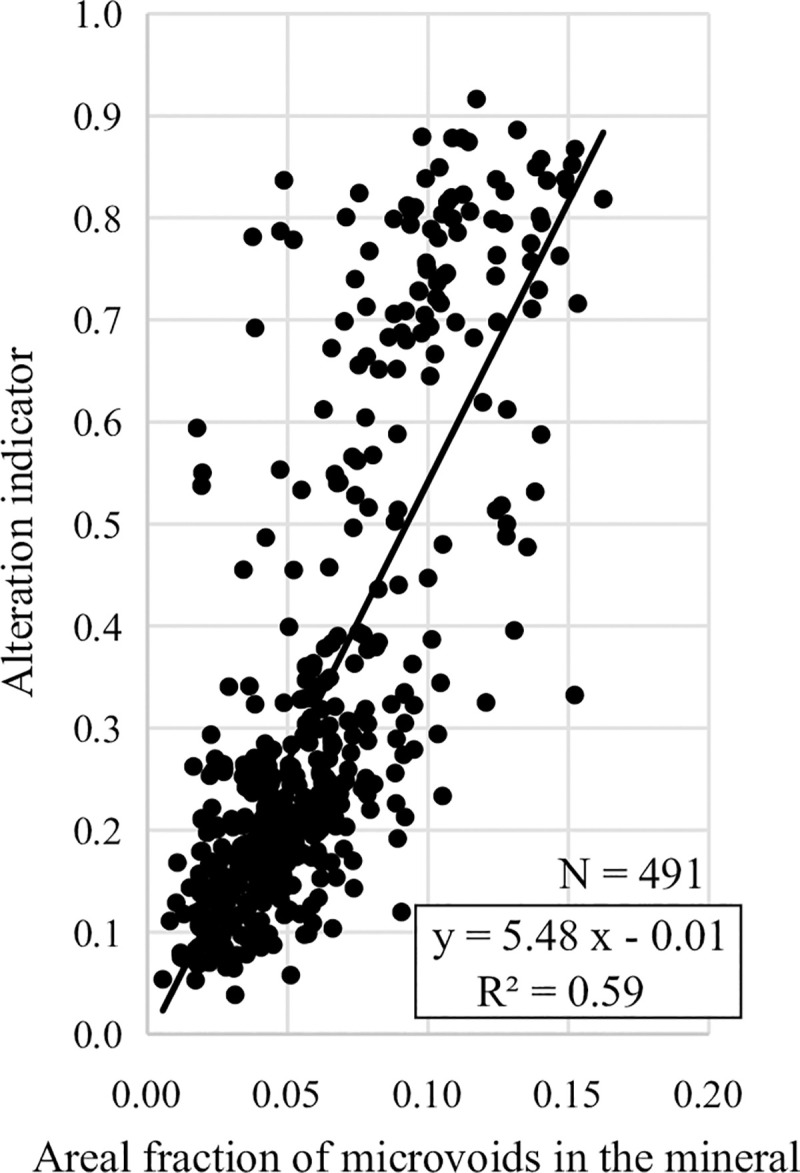
Relationship between the areal fraction of microvoids (microscopic fractures and micropores) in the chloritized biotites and the corresponding alteration indicator (N = 491).

**Fig 8:**
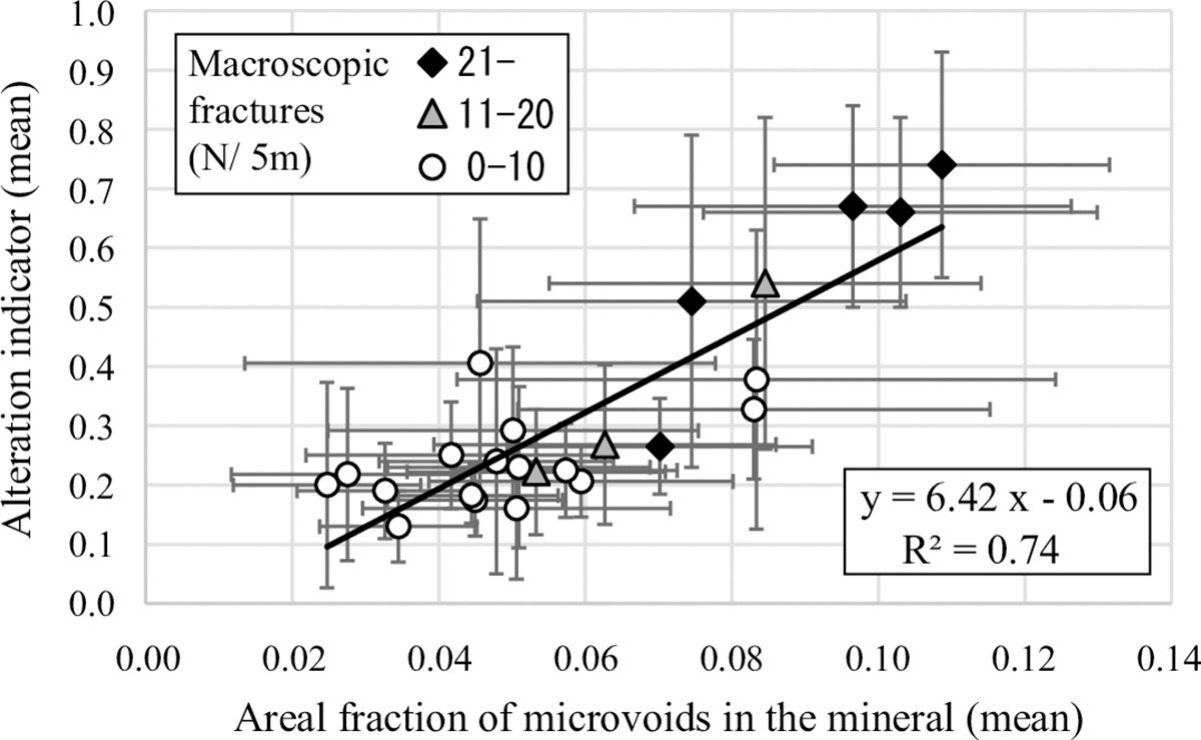
Relationship between the areal fractions of microvoids in the chloritized biotites (mean values for rock samples) and the corresponding alteration indicators (N = 24). Black diamond symbols denote samples with macroscopic fracture frequencies exceeding 21 fractures per 5 m. Grey triangle symbols are samples ranging from 11 to 20 fractures per 5 m in macroscopic fracture frequency, and open circle symbols indicate samples with macroscopic fracture frequencies below 10 fractures per 5 m.

Fig 7 shows the relationship between the areal fractions of microvoids in the chloritized biotites and the corresponding alteration indicators. Low areal fractions of microvoids correspond to small alteration indicators, and high areal fractions correspond to large indicators and demonstrate positive correlations: y = 5.48x – 0.01 and R^2^ = 0.59, where x is the areal fraction and y is the alteration indicator. Data above an alteration indicator of 0.4 are accompanied by smaller areal fractions relative to the regression line (Fig 7). Petrographic observations show that the biotite chloritization is accompanied by the precipitation of secondary minerals (e.g., carbonate minerals) within the microvoid with an increase in the extent of alteration [23]. This phenomenon causes a reduction in the areal fractions of microvoids in the chloritized biotite, resulting in a reduction in the correlation coefficient value in Fig 7. Fig 8 shows the relationship between the areal fractions of microvoid (x) and the alteration indicators (y) in rock samples, which also demonstrate strong positive correlations: y = 6.42x – 0.06 and R^2^ = 0.74. The development of hydrothermal alteration appears to be related to the areal fraction of microvoids. In other words, the volumes of the microscopic fractures and micropores in the mineral significantly constrain the developmental extent of hydrothermal alteration.

### Relationship among alteration indicators, microvoids, and macroscopic fractures

Fig 8 displays the relationship between the areal microvoid fractions, the alteration indicators in the rock samples, and the macroscopic fracture frequencies at the same intervals (S2 Table). Low macroscopic fracture frequencies correspond to low areal microvoid fractions, whereas small alteration indicators and high macroscopic fracture frequencies correspond to high areal fractions and large indicators (Fig 8). Therefore, the volume of microvoids and hydrothermal alteration development are related to the macroscopic fracture frequency.

### Genesis and development processes of fractures in granite

There is a positive correlation between the areal fractions of microvoids in minerals and the corresponding alteration indicators (Fig 7). The hydrothermal alteration of granitic rock is predominately constrained by dissolution–precipitation processes during the penetration of hydrothermal fluids through microvoids [23–25]. Therefore, microscopic fractures and micropores occurred before hydrothermal alteration. The volume of microscopic fractures and micropores places a significant constraint on the areal development of hydrothermal alteration.

The frequency of macroscopic fractures can be determined by the areal microvoid fractions in minerals (Fig 8), indicating that macroscopic fractures originated from microscopic fractures and micropores. Microscopic fractures and micropores occurred at the thermal stage between 350 and 780°C, which was deduced from two lines of evidence: 1) microscopic fractures and micropores never occurred at temperature conditions above the solidus (approximately 780°C [5]) and 2) they occurred before hydrothermal alteration [23–25]. The alteration processes of biotite chloritization occurred at temperatures of 180–350°C [23], i.e., in cooling granite, while the microvoids occurred at temperatures above 350°C (alteration onset temperature). The thermochronological evaluation corresponds to the thermal conditions from the solidus (approximately 780°C) to biotite K–Ar closure (350–400°C) [21]. The LCI was deduced from the development of sub-solidus reaction textures, which occur at the temperature range between 780°C and below 500°C during cooling [5]. Thermochronological evaluation and the LCI are strongly related to the frequency distribution of macroscopic fractures [5, 21], confirming that the microvoids occurred at temperatures between 350 and 780°C. The intersection of the *t-T* path (Fig 2) and thermal condition of 350–780°C yields a period of ca. 70–75 Ma.

The distribution of macroscopic fracture orientations is controlled by extrinsic factors, such as unloading and faulting (Fig 6), indicating that macroscopic fracture development occurred during the tectonic unloading and faulting event. Nishimoto et al. [22] reported that the faults within the Toki granite occurred during 18–12 Ma. The intersection of the *t-T* path (Fig 2) and temporal condition of 18–12 Ma yields about 30–80°C. Unloading was related with the exhumation process of the rock body [17, 41]. The Toki granite underwent exhumation during the period between ca. 50 Ma and the present [42]. The intersection of the *t-T* path and temporal condition of 50 Ma yields about 100–200°C. Therefore, unloading and faulting occurred after the formation of microscopic fractures and micropores, as well as hydrothermal alteration. Hence, in the cooling process of the granite, microscopic fractures and micropores within the minerals acted as a source of macroscopic fractures; subsequent tectonics (extrinsic factor) developed the microscopic fractures into macroscopic fractures. Therefore, intrinsic factors are used to evaluate the source of macroscopic fractures, thus contributing to an evaluation of the present and future frequency distribution of macroscopic fractures.

## Conclusion

The genesis and development processes of fractures in granite were described using the Toki granite in central Japan, for which we effectively characterized the fracture frequency distribution. In granitic rock, macroscopic fractures act as conduit pathways for fluid flow and contaminant transport. Appropriate characterization of the frequency distribution of macroscopic fractures in granite can assist in understanding potential hydrogeological applications, which contributes to safety evaluations for geological disposal and storage. We proposed petrographic alteration indicators using biotite chloritization as new methods to evaluate the extent of hydrothermal alteration and fracture frequency within granites. The alteration indicators are defined as the ratio between the alteration product area and the original mineral area. The volume of microscopic fractures and micropores in the mineral was quantitatively characterized by the areal fraction of microvoids in the minerals through image analysis. The technical developments of methodologies that characterize the alteration extent and areal fraction of microvoids are innovative for petrology and geology studies focusing on hydrothermal alteration. There were positive relationships among the 1) areal fractions of microvoids (microscopic fractures and micropores) in minerals, 2) corresponding alteration indicators, and 3) the macroscopic fracture frequencies at corresponding intervals in the Toki granite. Samples with low macroscopic fracture frequencies correspond to low areal microvoid fractions, whereas small alteration indicators and samples with high macroscopic fracture frequencies correspond to high areal fractions and large indicators.

The frequency of macroscopic fractures can be determined by the areal microvoid fractions in minerals, indicating that microscopic fractures and micropores act as the source of macroscopic fractures. In the cooling process, microscopic fractures and micropores occurred at the thermal stage between the solidus (approximately 780°C) and 350°C. Hydrothermal alteration occurred between 180 and 350°C. The volume of microvoids in the mineral place a significant constraint on the areal development of hydrothermal alteration; thus, microvoids can be evaluated by the alteration indicators. Unloading and faulting occurred after the formation of microscopic fractures and micropores, as well as hydrothermal alteration. The distribution of the macroscopic fracture orientations is controlled by extrinsic factors, such as unloading and faulting, indicating that macroscopic fracture development occurred during the tectonic event. Therefore, intrinsic factors, including the alteration indicators, can evaluate the volume of microscopic fractures and micropores (i.e., the source of macroscopic fractures), also contributing to an evaluation of the present and future distribution of macroscopic fracture frequencies.

## Supporting information

S1 Table Fractures of the 06MI03 borehole. ^a^mabh: meters along borehole. ^b^masl: meters above sea level. ^c^Frequency of macroscopic fracture. ^d^Samples are named as No. 1–12 and A1-A12. ^e^Upper highly fractured domain (UHFD) and lower sparsely fractured domain (LSFD). ^f^Hornblende biotite granite (HBG) and biotite granite (BG).
(XLSX)

S2 Table Alteration indicators and areal fractions of microscopic fractures in the chloritized biotites for samples No. A1-A12 and No. 1–12 and the corresponding macroscopic fracture frequency in the 06MI03 borehole. ^a^mabh: meters along borehole. ^b^masl: meters above sea level. ^c^Number of measured minerals. ^d^Mean value of alteration indicator. ^e^Standard deviation. ^f^Mean areal fraction of microvoids in the alteration minerals. ^g^Upper highly fractured domain (UHFD) and lower sparsely fractured domain (LSFD). ^h^Hornblende biotite granite (HBG) and biotite granite (BG).
(XLSX)

S3 Table Pixels and area of alteration minerals in all chloritized biotites for samples No. A1-A12 and No. 1–12, leading to the alteration indicators. (XLSX)

S4 Table Pixels and area of microvoids in all chloritized biotites for samples No. A1-A12 and No. 1–12, leading to the areal fractions of microvoids. (XLSX)
